# Honeybee communication during collective defence is shaped by predation

**DOI:** 10.1186/s12915-021-01028-x

**Published:** 2021-05-25

**Authors:** Andrea López-Incera, Morgane Nouvian, Katja Ried, Thomas Müller, Hans J. Briegel

**Affiliations:** 1 Institute for Theoretical Physics, Universität Innsbruck, Innsbruck, 6020 Austria; 2 Department of Biology, Universität Konstanz, Konstanz, 78457 Germany; 3 Zukunftskolleg, Universität Konstanz, Konstanz, 78457 Germany; 4 Centre for the Advanced Study of Collective Behavior, Universität Konstanz, Konstanz, 78457 Germany; 5 Department of Philosophy, Universität Konstanz, Konstanz, 78457 Germany

**Keywords:** Honeybee, Alarm pheromone, Collective behaviour, Evolution, Artificial intelligence

## Abstract

**Background:**

Social insect colonies routinely face large vertebrate predators, against which they need to mount a collective defence. To do so, honeybees use an alarm pheromone that recruits nearby bees into mass stinging of the perceived threat. This alarm pheromone is carried directly on the stinger; hence, its concentration builds up during the course of the attack. We investigate how bees react to different alarm pheromone concentrations and how this evolved response pattern leads to better coordination at the group level.

**Results:**

We first present a dose-response curve to the alarm pheromone, obtained experimentally. This data reveals two phases in the bees’ response: initially, bees become more likely to sting as the alarm pheromone concentration increases, but aggressiveness drops back when very high concentrations are reached. Second, we apply Projective Simulation to model each bee as an artificial learning agent that relies on the pheromone concentration to decide whether to sting or not. Individuals are rewarded based on the collective performance, thus emulating natural selection in these complex societies. By also modelling predators in a detailed way, we are able to identify the main selection pressures that shaped the response pattern observed experimentally. In particular, the likelihood to sting in the absence of alarm pheromone (starting point of the dose-response curve) is inversely related to the rate of false alarms, such that bees in environments with low predator density are less likely to waste efforts responding to irrelevant stimuli. This is compensated for by a steep increase in aggressiveness when the alarm pheromone concentration starts rising. The later decay in aggressiveness may be explained as a curbing mechanism preventing worker loss.

**Conclusions:**

Our work provides a detailed understanding of alarm pheromone responses in honeybees and sheds light on the selection pressures that brought them about. In addition, it establishes our approach as a powerful tool to explore how selection based on a collective outcome shapes individual responses, which remains a challenging issue in the field of evolutionary biology.

**Supplementary Information:**

The online version contains supplementary material available at (10.1186/s12915-021-01028-x).

## Background

Whoever has delighted in honey knows how much of a valuable food source a honeybee colony can be. To fend off the many predators attracted by this nutrient trove, honeybees have evolved stingers and a powerful venom efficient against vertebrates and invertebrates alike. But arguably their most important weapon is number: honeybees build a collective defence against intruders, stinging, threatening and harassing them in dozens or hundreds. Central to this response is the alarm pheromone carried directly on their stingers, whose banana-like scent is well known to beekeepers. When released, the sting alarm pheromone (SAP) alerts and attracts other bees, recruiting them to the site of the disturbance and priming them to sting. It is a chemically complex blend of over 40 compounds, but its main component, isoamyl acetate (IAA), is sufficient to trigger most of the behavioural response. The SAP has been extensively studied, both from the releaser end (production, dispersal) and from the recipient end (detection, reaction, role of the different compounds, role of the context in which it is perceived — reviewed in [1]). Despite this wealth of knowledge on the SAP, two important aspects of its function remain elusive: its quantitative action and the evolution of this response. In this study, we use a combination of in vivo and in silico methods to better understand how honeybees react to different concentrations of alarm pheromone, how this impacts the organisation of the collective response, and which selection pressures might have driven the evolution of this defensive strategy.

If they detect a threat, guard bees can disperse the SAP actively by raising their abdomen, extruding their stinger and fanning their wings. Alternatively, since the SAP is carried on the stinger itself, it is automatically released upon stinging. Thus, the SAP potentially carries information about the presence and location of a threat, but also about the magnitude of the attack already mounted against it. Several studies already demonstrated that the intensity of the response is correlated with the amount of alarm pheromone in the atmosphere [2–5]. While these studies provided valuable information, they all tested bees in groups — and often in field conditions; hence, they could not establish an individual dose-response curve. Furthermore, in many cases, the behavioural readout was rather indirect (e.g. attraction or fanning); only one study [5] actually measured stinging frequency. To comprehend how each bee makes the decision to sting, and thus how the colony as a whole coordinates actions during a defensive event, an individually resolved dose-response curve is necessary. Here, we took advantage of an assay developed a few years ago [6], which measures stinging responses under controlled conditions, to fill this knowledge gap. We found that, indeed, there is a steep ramp-up phase at low to medium alarm pheromone concentrations, in which the stinging likelihood of a bee increases together with the concentration. In addition, we show for the first time the existence of a second, decreasing phase at high pheromone concentrations. This is consistent with an anecdotal report that a high dose of IAA became repellent to bees [7].

How to interpret this experimental curve? More precisely, what could have driven the evolution of such a response pattern? In social insect colonies, individuals coordinate their actions to reach fitness goals set at the colony level, effectively functioning as a single evolutionary unit. Thus, individual responses can only be understood through the collective outcome that they contribute to. Making the link between individual and collective behaviour has been the focus of a large body of work. Modern tracking technologies [8] combined with physics-inspired modelling of individuals have proven that collective movement, for example of marching locusts and schooling fish [9–11], could arise from simple interaction rules between members of a group [12]. To explain the more complex division of labour of social insects, threshold models for stimulus-response reactions of individual agents have been used, but they usually do not give an account of the underlying mechanisms or of their evolution [13]. Such an evolutionary perspective on self-organisation has been provided using neural networks [14, 15] but while being one of the most powerful tools of machine learning, neural networks are typically hard to interpret because of their high dimensionality. Evolutionary game theory has also recently been applied to study task allocation in social insects [16], modelling behavioural changes on the scale of colony lifetime under certain imposed pay-off relations for the individual behaviour. Game-theoretic approaches provide interesting novel perspectives on the dynamics of task distribution in a population, but usually give no account of the agent-based mechanisms that underlie this dynamics [17], in stark contrast to the approach we will follow in this paper.

To address this evolutionary question, we resort to Projective Simulation [18], which is a simple model of agency that integrates a notion of episodic memory with a reinforcement learning mechanism. Projective Simulation (PS) allows for a realistic encoding of the sensory apparatus and motor abilities of the agents, which can perceive, make decisions and act as individuals. An important distinction between PS and classic neural network approaches is the straightforward interpretability of the decision process, because of its restricted dimensionality. When interacting with other agents, individual actions may influence the perceptions and responses of the rest of the ensemble, which in turn leads to the emergence of collective behaviour. Crucially, neither the individual responses nor the interaction rules among agents are fixed in advance. Instead, they are developed throughout a learning process in which the agents’ decisions are reinforced if they are beneficial under certain evolutionary pressures (note that here “learning” is therefore a process of selection between generations rather than happening within an individual’s lifetime). All of these features make Projective Simulation a suitable framework to model behavioural experiments like the one presented above, since it allows us to analyse the observed responses from both the individual and the collective perspectives. Furthermore, we are able to draw conclusions about the possible evolutionary pressures that may have led to such behaviour by means of the reinforcement learning process.

In this work, we model each bee as a learning PS agent and the colony as an ensemble of agents that undergoes repeated encounters with predators. During each encounter, bees can die from stinging but this also participates in deterring the predator, or they can be directly killed by the predator. Since the success of a bee colony is highly dependent on its available workforce, the overall performance of the colony is then evaluated by counting the number of bees that are still alive at the end of the encounter, and the individual response pattern is rewarded accordingly. Hence, the whole simulation is similar to an evolutionary process, in which the behaviour of each generation of bees is passed on depending on its reproductive success. By investigating systematically the parameters of a simple but realistic predator model, we found that the initial ramp-up in stinging responsiveness was mainly driven by uncertainty on the predator detection (frequency of false alarms) and that the pheromone concentration at peak aggressiveness was dependent on the most resistant predator encountered. The second, decreasing phase could be interpreted as the combination of a self-limiting mechanism to avoid over-stinging (i.e. stinging far beyond what would be necessary to deter the predator) and of a return to baseline due to lack of experience in this range of concentrations.

The native range of the Western honeybee (*Apis mellifera*) spans a large part of Europe, Africa and Middle East Asia [19] and thus includes a wide diversity of ecosystems. As a consequence, multiple subspecies exist that have adapted to local conditions. In particular, the African honeybees are known for having stronger defensive reactions than their European counterparts: they recruit more bees, do so more quickly and are more persistent [20, 21]. Part of the explanation resides in their higher sensitivity to the SAP [4]. As a final test of our model, we tune the parameters to represent the constraints that were hypothesised to drive the evolution of this striking difference in behaviour (mainly a higher predation rate in Africa). We show that, with this input, our model accurately predicts the different strategies adopted by each subspecies. Thus, this novel application of Projective Simulation [22] to a group of agents with a common goal is promising for the study of social insects in general, and of the honeybee defensive behaviour in particular.

## Results

### Experimental results

**Fig. 1.**
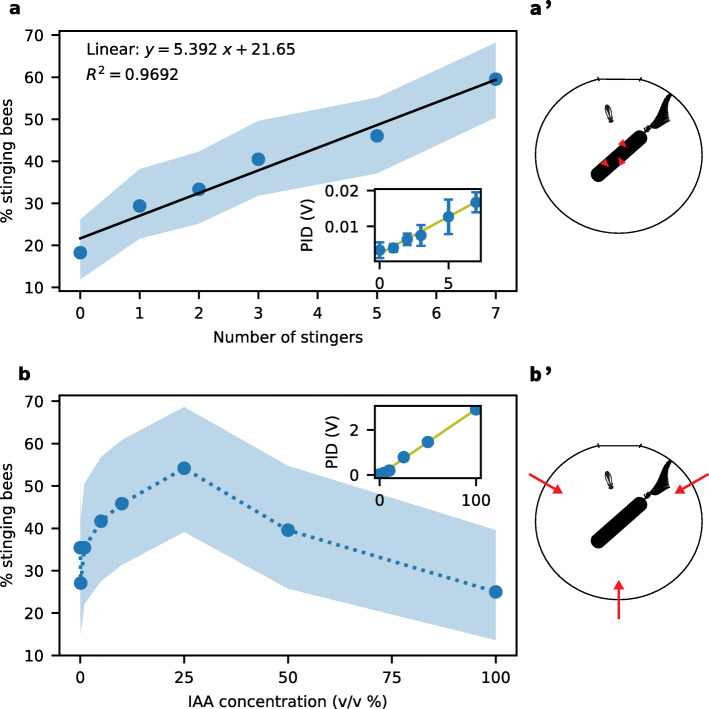
Stinging response as a function of the pheromone concentration. **a** Stinging frequency of a bee as a function of the number of pulled stingers on the dummy. **a’** Schematic of the behavioural assay (top view). The bee is facing a rotating dummy inside the arena, on which pulled stingers (red arrowheads) have been placed. **b** Stinging frequency of a bee as a function of the IAA concentration inside the arena. **b’** Schematic of the behavioural assay (top view). The bee is facing a rotating dummy inside the arena, while the alarm pheromone is carried in by three air flows (red arrows). In both graphs, the shaded area corresponds to the 95% confidence interval, estimated from a binomial distribution with our sample size (126 and 72 bees for each point in a and b, respectively). Insets show photoionization detector (PID) measurements of the SAP and IAA concentrations, which are linearly correlated with both the number of stingers and the dilution factor

To better understand if and how bees use the alarm pheromone concentration as a source of information during a defensive event, we first sought to establish a dose-response curve to the SAP. This requires to precisely control the pheromone concentration inside the testing arena. We used two methods to create a range of alarm pheromone concentrations: (1) pulling out a defined number of stingers from live, cold-anaesthetised bees, or (2) diluting synthetic IAA. To verify that the final concentrations scaled linearly with either the number of stingers or the dilution factor, we measured odour concentrations inside the arena using a photoionization detector (PID). As shown on Fig. 1, both methods reliably created linear series of concentrations (IAA: Pearson’s *r*=0.9989,*p*<0.001; stingers: Pearson’s *r*=0.9922,*p*<0.001). The absolute concentrations reached by using stingers appear to be much lower than the ones obtained with synthetic IAA, but one should keep in mind that the delivery method was also very different (stingers placed on the dummy vs. IAA carried in by the air flow) and that only a subset of the SAP compounds can be detected by the PID, making a direct comparison difficult.

**Fig. 2.**
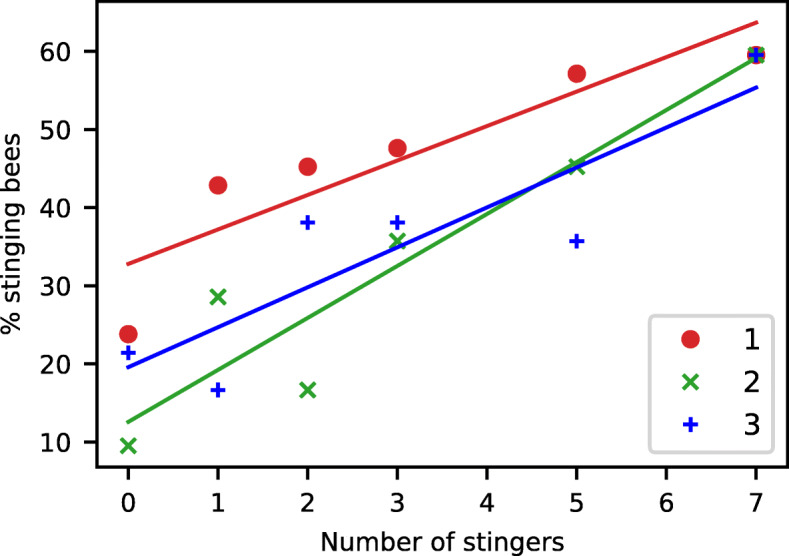
Stinging frequency of a bee as a function of the number of pulled stingers on the dummy, for different colonies of origin (numbered from 1 to 3), evaluated using *n* = 42 bees for each data point, i.e. a total of 756 bees across the 3 colonies. An ANOCOVA detected no significant difference in alarm pheromone responsiveness between colonies (similar slopes), but bees from colony 1 were more aggressive than bees from colony 2 (different means)

We first evaluated the stinging response of single bees faced with a rotating dummy on which a certain number of freshly pulled stingers were placed, to mimic previous attacks by other bees. The stinging likelihood of an individual bee increased linearly with the number of stingers (see Fig. 1a), from about 20% of the bees reacting to the dummy alone to 60% of them stinging when 7 stingers — and hence 7 “units” of SAP — were added (*n*=126 bees per data point so 756 bees in total; Pearson’s *r*=0.9845,*p*<0.001). Three colonies contributed equally to this dataset, which allowed us to check for variations in this response pattern (see Fig. 2). We found no significant difference on the regression slope between colonies (ANOCOVA, interaction term: *F*(2,754)=0.9,*p*=0.432), indicating that the effect of the SAP was similar on all bees. However, bees from colony 1 were overall more likely to sting than bees from colony 2 (ANOCOVA followed by Tukey’s HSD on intercepts, *p*=0.024), while bees from colony 3 showed intermediate aggressiveness (ANOCOVA followed by Tukey’s HSD on intercepts, 1 vs. 3: *p*=0.150, 2 vs. 3: *p*=0.550). Such behavioural variability is not surprising, as it is known that the aggressiveness of honeybees is strongly influenced by genetic factors [23].

The advantage of getting the SAP from stingers is that we could work with the full pheromonal blend, which is otherwise difficult to obtain. Its main inconvenience, however, is that only limited concentrations can be reached. To get at these higher concentrations, we repeated the experiment with different dilutions of IAA, the main active component of the SAP. The results are shown in Fig. 1b. Consistent with our previous results, we observed a linear increase in stinging responsiveness between 0 and 25% IAA (Pearson’s *r*=0.9127,*p*=0.011). The two higher concentrations that we sampled (50% and 100%) revealed a decay in aggressiveness. While these 3 points were not sufficient to test for a correlation, the decrease in stinging frequency between 25 and 100% was significant (*χ*^2^(1,96)=7.3612,*p*=0.007).

### Modelling the honeybees’ collective response

**Fig. 3.**
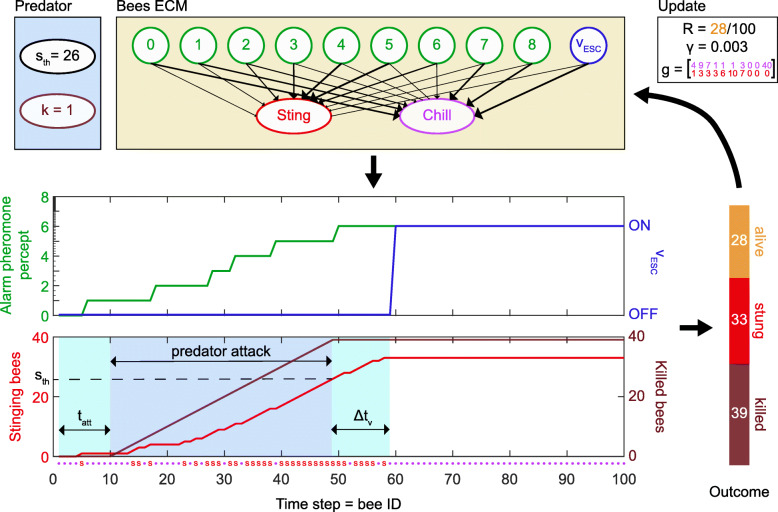
Theoretical model of one defensive event. We model the colony as an ensemble of *N*=100 identical bees, which are artificial learning agents that decide whether to sting (“Sting” and *S*) or not (“Chill” and ∙) based on their sensory perception. These percepts include the alarm pheromone concentration (binned logarithmically from 0 to 8) and a visual signal that the predator is leaving, *v*_ESC_. The 100 bees act sequentially, so there are 100 time steps, and each stinging bee releases one unit of alarm pheromone (small ticks on middle panel’s *y* axis), so that the sensory environment of a bee is defined by the behaviour of previous bees. A predator attacks the colony and kills *k* bee per time step from the time it reaches the colony, *t*_*att*_, until it receives a certain number of stings *s*_*th*_. At this point, it stops killing, but is still in the vicinity for *Δt*_*v*_ time steps before truly escaping, modelled as the activation of *v*_ESC_ for the remaining bees after time *Δt*_*v*_. Once every bee has made a decision, the outcome of the defensive event is evaluated and the individual decision process is updated based on the colony performance (reward factor *R*, proportional to the number of remaining live bees) for each percept (glow matrix *g*), with some forgetting (*γ*). The upper panels show the internal structure of bees and the predator’s parameters, the middle panel the time course of the bees’ perception and the bottom panel the behaviour of both bees and predator during an example trial

The agent-based learning framework of PS [18] was used to model the defensive behaviour of honeybee colonies. PS agents possess an explicit episodic memory (ECM), which is a network of clips representing how sensory information encountered by the agent is connected with possible action outputs (Fig. 3). The state of the agent’s memory (i.e. the weight of the connections) at a given time is represented by a real-valued matrix, *h*. In our case, this memory structure is not acquired through individual experience, but rather selected across generations. This evolutionary process is represented by the parameters *g*, *R* and *γ*, which model, respectively, the past behaviour of the population, the success of the current strategy and “forgetting” (imperfect selection). To represent different environmental pressures that may shape the resulting collective strategy, we also consider that bee populations may vary in how early they detect a predator approaching (*t*_*att*_), and in the time lag *Δt*_*v*_ with which the bees visually perceive the predator’s desistance, enabling them to terminate their defence independently of pheromone percepts. Finally, our model includes several variables describing the predation pressure experienced by bee colonies. These are the killing rate *k* of a predator (number of killed bees per time step), a threshold value *s*_*th*_ quantifying the number of stings after which a predator stops its attack, and the rate of false alarms *r*_*f*_. A more detailed description of the model and its parameters is given in the “Methods” section and in Fig. 3.

In the following, we describe the variations of the model through which we tested the influence of different environmental pressures on the resulting collective strategy, with a view to uncovering a plausible causal explanation for the empirically observed reaction of bees to the alarm pheromone.

### Interval between predator detection and the start of its attack

A small proportion of honeybees, termed guards, sit at the nest entrance and monitor its surroundings [1]. They may detect predators early enough to start the defensive response before the intruder reaches the colony. We varied the time *t*_*att*_ at which a predator starts killing bees after it was detected: high values for *t*_*att*_ thus represent colonies that invest heavily in guards or monitor large areas, whereas low values can be taken to represent colonies that only get alerted once the predator is already close by. As shown in Fig. ??a, we find that the probabilities of stinging are lower when the bees detect the predator early (*t*_*att*_=60) than when they do not have guards (*t*_*att*_=0). Nonetheless, populations with guards actually fare better than their counterparts, as the predator has less time to kill bees before being deterred (Fig. ??a’).

**Fig. 4.**
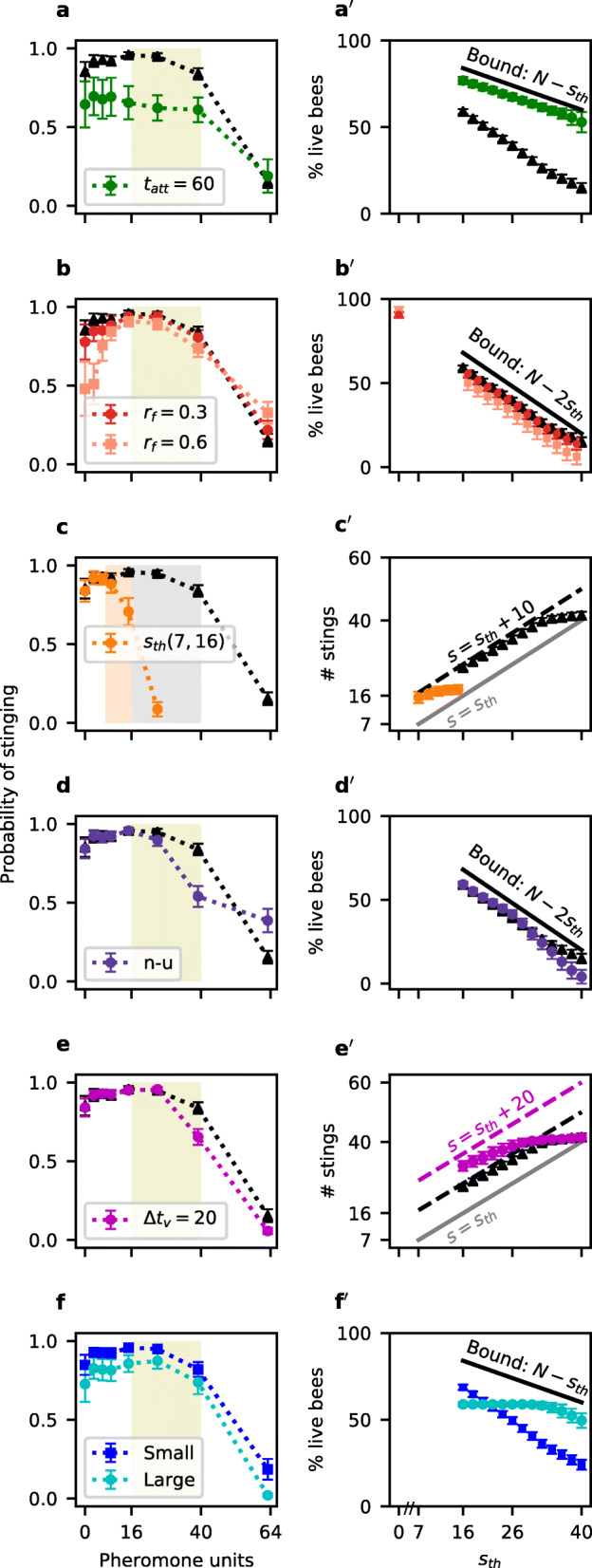
Stinging response as a function of alarm pheromone concentration (first column) and performance (second column) in modelled colonies facing different environmental pressures. Triangles denote colonies trained for 80,000 trials with parameters: *N*=100,*s*_*th*_∈(16,40),*k*=1,*t*_*att*_=0,*r*_*f*_=0 and *Δt*_*v*_=10. They are compared to colonies that **a** invest in guards to detect the predator earlier (*t*_*att*_=60); **b** have higher false alarm rates (*r*_*f*_=0.3,0.6); **c** face only weak predators (*s*_*th*_∈(7,16)); **d** face a non-uniform (n-u) distribution of predators, in which weak predators (*s*_*th*_∈(16,26)) appear 4 times more often than strong ones (*s*_*th*_∈(27,40)); and **e** need more time to visually detect that the predator is escaping (*Δt*_*v*_=20). Only one parameter is varied in each comparison. Panel **f** compares colonies that defend small and large territory areas, which we model by setting *t*_*att*_=*Δt*_*v*_=10 and *t*_*att*_=*Δt*_*v*_=40, respectively. In all plots of the first column, percepts in which the probability of stinging remained as initialised (*p*_*s*_=0.5) because they were never reached are not included. Shaded areas indicate the *s*_*th*_ range. Markers are at the end of each percept’s bin. Probabilities *p*_*s*_ for percept *v*_ESC_ are given in Table 1. Average ± one standard deviation for 50 independently trained populations. In the second column, panels **a’**, **b’**, **d’** and **f’** display the percentage of live bees at the end of encounters, depending on the predator resistance *s*_*th*_. The upper bound indicates the optimal performance for the given scenario. Panels **c’** and **e’** show the total number of bees stinging as a function of predator resistance, and the dashed lines indicate the maximum number of times bees could sting before percept *v*_ESC_ is activated. Numbers below this boundary indicate self-limitation based on pheromone concentration. Average ± one standard deviation in the last 500 trials of 50 independently trained populations

### Predation rate

In our model, each trial corresponds to a defensive event, which we assume is started by a non-specified disturbance close to the colony (for example, the visual perception of an object moving in the vicinity). In reality, most of these stimuli are likely to be unrelated to predation: they could be animals just passing by, falling branches and so on. Reacting to these stimuli would mean that bees waste time and effort that could be better invested (and, at worst, even die from stinging unnecessarily). The frequency of these *false alarms* depends on the environment considered: a high density of predators and/or the presence of specialised predators may be translated by a high predation rate for colonies, and hence fewer false alarms. To explore this, we include in our model trials in which there is no predator, which appear with probability *r*_*f*_. Formally, for these trials, *s*_*th*_ is set to 0, since there is no actual need to sting. We observe that, as the percentage of false alarms in the learning process grows larger, the initial probability of stinging gets lower but the maximum reached stays similar (Fig. ??b). This ramp-up is consistent with the experimental data and can be interpreted as follows: since overreaction has a cost for the colony, few bees would sting in the absence of alarm pheromone, which prevents many bees from leaving the hive after every minimal signal of danger. However, the pronounced increase in the probability of stinging (*p*_*s*_) enables a fast recruitment when there actually is a predator. Hence, the steepness of the ramp-up phase gives us insights into the predation rate to which populations were subjected.

**Table 1:** Probability of stinging (*p*_*s*_) for percept *v*_ESC_ at the end of learning processes with the parameters specified that are analysed in Fig. ??. Learning process #1 (triangles in Fig. ??) is used as a baseline for comparison

		Parameters					
Panel	Process #	*s*_*th*_	*k*	*r*_*f*_	*t*_*att*_	*Δt*_*v*_	*p*_*s*_±std
a	1	(16,40)	1	0	**0**	10	0.005±0.002
	2	(16,40)	1	0	**60**	10	0.023±0.011
b	1	(16,40)	1	**0**	0	10	0.005±0.002
	3	(16,40)	1	**0.3**	0	10	0.012±0.006
	4	(16,40)	1	**0.6**	0	10	0.024±0.010
c	1	**(16,40)**	1	0	0	10	0.005±0.002
	5	**(7,16)**	1	0	0	10	0.018±0.005
d	1	**(16,40)**	1	0	0	10	0.005±0.002
	6	**(16,40) n-u**	1	0	0	10	0.007±0.003
e	1	(16,40)	1	0	0	**10**	0.005±0.002
	7	(16,40)	1	0	0	**20**	0.006±0.002
f	8	(16,40)	1	0	**10**	**10**	0.007±0.003
	9	(16,40)	1	0	**40**	**40**	0.024±0.014

Only one parameter (indicated in bold) is varied in each comparison. See main text for details. *n-u* non-uniform distribution

### Diversity of predators

Honeybee colonies attract a wide array of vertebrate predators, from mice and toads to humans and bears. Obviously some are easier to deter than others, and their distribution may vary depending on the ecosystem considered. We evaluate the influence of predator diversity by changing the range of *s*_*th*_. Each population adapted primarily to the most resistant predator encountered, with bees stinging with very high probability until they reach an alarm pheromone concentration at which all predators should be gone (Fig. ??c). While it makes sense that honeybee colonies need to be able to cope with the worst predator in their environment, it is interesting to note that “weak” predators have little impact on the defensive strategy adopted. Predators can also vary in the relative frequency at which they are encountered. We investigate this by comparing a scenario with uniform distribution of predators to one in which “small” (*s*_*th*_∈(16,26)) predators are 4 times more likely than “large” (*s*_*th*_∈(27,40)) ones. We observe (Fig. ??d) that the bees that more often encounter weak predators have a lower probability of stinging at high pheromone concentrations, most likely because these are not often reached. As a result, these populations are less efficient against large predators (Fig. ??d’). Hence, the relative abundance of the different predators in a given environment also influences the defensive strategy adopted.

### Defence termination

Finally, we consider how bees determine when to *stop* stinging. Realistically, a predator does not disappear as soon as its stinging threshold is reached: it needs time to move outside of the defended area. This delay is implemented as the parameter *Δt*_*v*_ in our model. Agents trained with *Δt*_*v*_=20 have lower probabilities of stinging than those with *Δt*_*v*_=10 for high pheromone concentrations (Fig. ??e). Since it takes more time for the predator to leave, these populations are at a higher risk of “wasting” bees (i.e. bees stinging even though *s*_*th*_ was already reached), as is indeed observed for weak predators. However, they seem to compensate this effect when dealing with strong predators by relying on high pheromone concentrations to signal that an efficient defensive response has already been achieved. Thanks to this adaptation, they are able to curb their number of stings to value close to *s*_*th*_ for strong predators (Fig. ??e’). This could explain the decay in alarm pheromone responsiveness observed in the experimental data. Alternatively, this decay may be a simple return to baseline, as is observed when the agents do not have to address this issue (*Δt*_*v*_=0, the predator is immediately removed, see Additional file 1: Figure S1). In any case, measuring when this decay starts would provide information about the range of predators encountered by real populations.

### Territory size

Both *t*_*att*_ and *Δt*_*v*_ are linked to the time needed for the predator to move from the edge of the defended territory to the nest itself. Hence, we could expect that for a given population, *t*_*att*_=*Δt*_*v*_ and that this single value represents the territory radius. It is interesting to note that an optimal behaviour would require a high value for *t*_*att*_ (to get a better chance to cope with predators with high *s*_*th*_, Fig. ??a’) but a low value for *Δt*_*v*_ (to avoid over-stinging predators with small *s*_*th*_, Fig. ??e’). This is indeed what we observe in Fig. ??f’: colonies defending a smaller area perform better when confronted with weak predators, but lose more bees when faced with resistant predators. Thus, defending a large area seems to be an adaptation to predators that are on average quite resistant, whereas defending a small area is a better strategy when most predators can be easily deterred.

### Case study: European vs. African bees

**Fig. 5.**
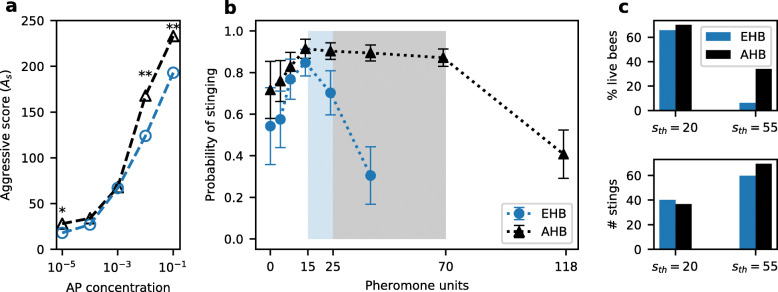
Comparison of European and African(ized) honeybees. **a** Comparison of the aggressive score as a function of the alarm pheromone concentration for Africanized (AHB, black triangles) and European (EHB, blue circles) honeybees. Experimental data modified from [4] (see the “Comparison between Africanized and European bees” section). Stars indicate significant differences in responsiveness according to the original paper (* *p*<0.05, ** *p*<0.01). **b** Comparison of the learned probabilities of stinging between European and African populations, from modelling. Shaded areas indicate the range of predators that European (blue, *s*_*th*_∈(15,25)) and African (grey, *s*_*th*_∈(15,70)) colonies faced during the learning process. Average ± one standard deviation from 50 independently trained populations, at the end of a learning process with 10^5^ trials. For clarity, percepts for which the probability of stinging remains at the initialisation values (*p*_*s*_=0.5) are not shown. Visual percept *v*_ESC_: *p*_*s*_=0.09±0.01 for EHB and *p*_*s*_=0.05±0.01 for AHB. Parameters: $N=200, \gamma =0.003, k=1, t_{{att}}=0, \Delta t_{v}=10, r_{f}^{E}=0.6, r_{f}^{A}=0.3$. **c** Performance of each colony when faced with predators of sizes *s*_*th*_=20,55, from modelling. European colonies go extinct when they encounter a predator that is more resistant than the ones they faced during the learning process, whereas African colonies are able to survive

The native range of the Western honeybee (*Apis mellifera*) includes a wide variety of ecosystems [19]. As a consequence, multiple subspecies exist that have adapted to local conditions. Among them, *A. m. scutellata* (which we call here “African bees” for simplicity) are well known for their fierce attacks, although most of the experimental data was gathered on “Africanized” bees, a hybrid subspecies which retained the defensive behaviour of their African ascendants. When faced with a predator, these bees recruit more bees, do so more quickly and are more persistent [20, 21]. Part of the explanation could reside in their higher sensitivity to the SAP, as shown in Fig. 5a (data from [4]). It has been hypothesised that several traits of African bees, including high defensiveness, evolved in response to higher predation rates in the tropics [24, 25]. From our model perspective, this would mean fewer false alarms. African bees may also face specialised predators such as ratels (*Mellivora capensis* [26–28]), which are more difficult to deter. In our model, we translate this into a higher range of *s*_*th*_ for African bees than for European bees.

We modelled two types of bee populations that differed only with respect to these properties: frequency of false alarms (*r*_*f*_) and range of *s*_*th*_. Consistent with experimental data, the populations which have learned to defend against larger and more frequent predators develop a stronger reaction to the pheromone. The higher frequency of attacks in the African case drives the agents to react more strongly at low concentrations. In addition, the probability of stinging remains high over a much larger range of alarm pheromone concentrations due to the larger *s*_*th*_ interval (Fig. 5b). As a result, African populations are able to survive attacks from very resistant predators, whereas European populations would go nearly extinct (Fig. 5c). These results support the hypothesis that the difference in aggressiveness at low pheromone concentrations [4] is due to higher attack rates in Africa. Furthermore, under the assumption that African bees also encounter more resistant predators, we observe that they keep stinging over a broader range of alarm pheromone concentrations. It would be interesting to test African(ized) bees at these higher pheromone concentrations, as we did for European bees, in order to verify this prediction.

## Discussion

We use experimental and theoretical work to better understand how the collective defensive behaviour of honeybees is organised. We show that not only the presence, but also the concentration of alarm pheromone provides important information to individual bees. We explore the meaning of this regulation of stinging behaviour by alarm pheromone concentration via a relatively simple model of collective defence and characterise the shaping role of several environmental factors such as predation rate, predator resistance and predator diversity.

The architecture of our agent’s decision-making process is very simple: it only postulates that the agent can discriminate between different concentrations of pheromone and acts accordingly. Honeybees are capable of assessing and even learning absolute odour concentrations [29]; hence, it would be tempting to see our model as a simplified view of the processing being implemented by an actual bee brain. However, we have to keep in mind that our experimental results were obtained by testing each bee only once and can thus be interpreted in another way: the gradation in response could arise at the population level, from varying thresholds of response between bees [30] (rather than within each individual bee). It is important to note that both interpretations are valid within the scope of our model, because our reward scheme is based on the population response. In the first interpretation, the internal structure of each agent could thus directly represent the decision-making process of a real bee, hard-wired in the brain. In the alternative interpretation, the probability of stinging would be more accurately described as the proportion of bees with a stinging threshold lower than the given alarm pheromone concentration, and hence, the internal structure of the agents would rather represent the decision process of an ersatz “average bee” given this population structure. In this case, selection should act on the mechanisms controlling this individual variability, to ensure that an appropriate distribution of thresholds is maintained across generations [31]. Further experiments would be necessary to decide between these two interpretations, for example by repeatedly testing individuals at different concentrations.

Independently of this interpretation, our data suggests that each individual has at least two internal thresholds: one to start stinging and one to stop. This is consistent with an anecdotal field observation that high concentrations of alarm pheromone become repulsive to bees [7]. Indeed, without a stopping threshold, the dose-response curve would only plateau at high concentrations instead of decreasing as we observed. The ecological function of this newly discovered second threshold, according to our theoretical results, could be to avoid over-stinging (i.e. stinging and chasing of a predator already in the process of moving away from the colony). Alternatively, it could be that these high concentrations are never reached in the wild and hence that no adaptive response could evolve.

Of course, our model is by no means exhaustive: there may be other environmental factors influencing the honeybees’ defensive behaviour that we did not investigate. We did try, however, to include the most common descriptors of predation. It is also important to note that the factors that we did include are sufficient to provide plausible explanations regarding the adaptive value of the alarm pheromone dose-response curve. Moreover, each of these factors only had a narrow effect on alarm pheromone responsiveness, such that the features of the experimental curve could not be obtained in many different ways. As a consequence, our model could successfully identify the most pertinent factors.

Detailed ecological surveys of the interactions between bees and predators are difficult to conduct, and indeed, we could not find any quantitative information about the prevalence of specific predators or about predation rates in a given environment. Our results could serve as an alternative tool to gather information on this topic. We provide an example of this using previously collected data comparing African(ized) and European bees and found that our model accurately predicted the experimental data when considering that African bees were subjected to higher predation rates. Hence, measuring alarm pheromone responses could inform about the natural history and environment of given populations where this knowledge is missing. Nonetheless, our results should first be validated by gathering accurate field data before they could be used in this way. From what we already know, African bees defend a much larger area around their nest [20]. According to our model, this strategy is better suited to tackle resistant predators. Future studies could also test this hypothesis by experimentally measuring the SAP concentration at which individual stinging responsiveness is maximum, since we found this to be representative of the most resistant predator.

On the theoretical side, our model could be expanded into a more fine-grained analysis of collective defence. For example, it would be interesting to allow some heterogeneity between agents, since we know that at least two types of bees participate in the defence: guards and soldiers [1]. Another interesting challenge would be to let the agents learn about the optimal size of the territory to defend (*t*_*att*_ and *Δt*_*v*_). Finally and most importantly, we believe that this agent-based modelling approach is an exciting tool to study the evolution of collective behaviour in general, which could be applied to other tasks and species. For example, trail building and following in ants is also a process that relies on pheromone accumulation and in which individuals make decisions based on pheromone concentration [32, 33]. It could be interesting to adapt our framework to this alternative type of collective decision-making.

## Conclusions

Social insect colonies are often called “superorganisms” because of how some tasks are distributed between colony members, which is reminiscent of the different functions of cells and organs in multi-cellular organisms. Understanding how such coordination is achieved and how selection shaped the behavioural responses of individual group members is a fascinating and complex question. Here, we contribute to this field of research by combining experimental work and a novel computational approach to better comprehend the collective defensive behaviour of honeybees. In particular, we focused on responsiveness to the sting alarm pheromone, as this signalling mechanism is at the core of the bees’ communication during a defensive event. First, we show experimentally that the stinging likelihood of individual bees varies depending on the concentration of SAP in the atmosphere. This response pattern exhibits at least two phases: an initial, ramp-up phase from low to intermediate concentrations of pheromone, followed by a decrease at high concentrations. To interpret these results, we built a relatively simple agent-based model of the honeybee defensive behaviour. The novelty of our approach resides in adapting Projective Simulation to a group of agents with a common goal (and hence a common reward scheme). We also added the constraint that all agents inherit the same decision process, as this better represents the heritability of aggressive traits across generations. This agent model allowed us to explore the impact of different evolutionary pressures on individual responsiveness to the alarm pheromone. From these insights, we postulate that the existence of the first phase (ramp-up) in SAP responsiveness results from a trade-off between avoiding false alarms and quickly recruiting nestmates in the presence of real predators. The SAP intensity at which the stinging probability peaks depends on the most resistant predator in a given environment. Finally, the decrease in stinging likelihood at high SAP concentrations could be due to a self-limiting mechanism to avoid unnecessary stings, or simply the consequence of a return to baseline because such high concentrations are never encountered in the wild, and hence no specific response had the chance to evolve. Altogether, our work provides new insights into the defensive behaviour of honeybees and establishes PS as a promising tool to explore how selection on a collective outcome drives the evolution of individual responses.

## Methods

### Experimental material and methods

#### Honeybees

The experiments were conducted at the University of Konstanz, with honeybees (*Apis mellifera*) from freely foraging colonies hosted on the roof. The experiment in which the alarm pheromone was obtained by pulling out stingers (see the “Alarm pheromone” section below) was conducted between May and August 2018. Three colonies contributed equally to this experiment (*n*=252 bees per colony, 756 bees in total). The experiment with synthetic alarm pheromone was conducted in May/June 2019. The bees were taken from 4 colonies (including one from 2018, the other colonies were lost during the winter), again in equal numbers (*n*=96 bees per colony, 384 bees in total). To catch the bees, a black ostrich feather was waved in front of the hive entrance. The bees attacking the feather (and thus involved in colony defence) were collected in a plastic bag and cooled in ice for a few minutes, until they stopped moving. They were then placed alone in a modified syringe with ad libitum sugar water (50% vol/vol) and given at least 15 min to recover from the cold anaesthesia before testing.

#### Alarm pheromone

The alarm pheromone of honeybees includes over 40 compounds [34, 35], making it difficult to synthesise. Hence, in a first experiment, we pulled stingers out of cold-anaesthetised bees to get the full alarm pheromone blend. This manipulation was done as fast as possible and just before the start of the trial. The stingers were placed on the dummy (the stinging target) to mimic other bees stinging it before the start of the trial. The range of alarm pheromone concentration was obtained by varying the number of stingers: 0, 1, 2, 3, 5 or 7 stingers. A clean air flow entered the arena from 3 holes on the sides, equally spaced, and the arena lid was drilled with an array of small holes to allow the air out. The advantage of pulling stingers was to obtain the full odour blend, but the inconvenience was that the concentrations we reached were limited. To cope with this issue, we performed a second set of tests in which we only used the main component of the alarm pheromone, iso-amylacetate (IAA, Merck *Ⓡ*). IAA is sufficient to reproduce most of the action of the full blend [36, 37]. In this case, IAA was diluted in mineral oil (Merck *Ⓡ*) to a final concentration (vol/vol) of 0% (control), 0.1%, 1%, 5%, 10%, 25%, 50% or 100% (pure). To deliver the odour, 10*μl* of solution were put on a small filter paper which was then placed inside the air flow entering the arena. To verify that the odour concentration was linearly correlated to either the number of stingers or the dilution ratio, measurements were made inside the arena with a photoionization detector (PID). The measures were taken every 0.01 s, and the data was smoothed on a sliding 1 s (101 points) time window centred on each point. The amplitude of the odour signal was then calculated by subtracting the baseline (average of the 5 s just before the stingers were inserted or the air flow was started) from the peak value (average of the 5 s centred on the maximum value reached). For tests with IAA, a single measure was taken for each concentration. In tests with stingers, the concentrations were close to the limit of the PID sensitivity; hence, we repeated the measures 3 times and averaged the results to increase reliability.

#### Stinging assay

The protocol for the stinging assay has been described in detail in [6]. Briefly, the bee was introduced into a cylindrical testing arena where it faced a rotating dummy coated in black leather. The stinging behaviour was first scored visually and defined by the bee adopting the characteristic stinging posture: arched with the tip of the abdomen pressed on the dummy. This was further confirmed at the end of the trial by the presence of the stinger, embedded in the leather.

#### Comparison between Africanized and European bees

It has been shown that Africanized bees are more sensitive to SAP [4]. In this previous study, the responses of caged honeybees to different concentrations of alarm pheromone were classified into 5 categories according to their intensity: “no response” (N), “weak response” (W), “moderate response” (M), “strong response” (S) or “very strong response” (V). To better visualise this data and to be able to compare it to our model results, we transformed this data by calculating an “Aggressive score” (*A*_*s*_) for each alarm pheromone concentration and for each ecotype, which was defined as *A*_*s*_=*N*×0+*W*×1+*M*×2+*S*×3+*V*×4, with each letter corresponding to the percentage of reactions that fell into the corresponding category.

### Theoretical model: Projective Simulation

Projective Simulation (PS) [18, 38–42] is a model for artificial agency that combines a notion of episodic memory with a simple reinforcement learning mechanism. It allows an agent to adapt its internal decision-making processes and improve its performance in a given environment. PS has a transparent structure than can be analysed and interpreted throughout the learning process. This feature is of particular importance in this work, since we aim at *explaining* the experimentally observed individual responses to certain stimuli.

In the context of behavioural biology, the model of PS offers the possibility of enriching the description of the entities’ sensorimotor abilities to get closer to the real mechanisms, which can help gain new insight into phenomena that too simplified or abstract models cannot account for. Honeybees offer an interesting opportunity for PS since they exhibit complex behaviours at both the individual and the collective level despite their relatively small brain. In addition, Projective Simulation can be used to model collective behaviour [22, 43] by considering ensembles of PS agents that interact with each other. In the present work, this interaction is determined by the olfactory perception of the pheromone that bees release when stinging. The fact that each agent has an individual deliberation process allows us not only to explain the experimental results but also to study how the individual responses to alarm pheromone are combined into an appropriate defensive reaction for the colony.

In this section, we describe the general features of Projective Simulation and we further specify how we model the scenario of colony defence in the “Details of the model I: the bee and the learning process” and “Details of the model II: the predator” sections.

The individual interaction of a PS agent with its surroundings starts with the agent perceiving some input information, which triggers a deliberation process that ends with the agent performing a certain action. The deliberation process is carried out by the main internal structure of the agent — called episodic and compositional memory (ECM) [18] —, which is a network consisting of nodes, termed *clips*, connected by edges. Clips represent snippets (or “episodes”) of the agent’s experience and can encode information from basic percepts, like a colour or an odour, to compositions of short sequences of sensory information. Each clip is connected to its neighbouring clips by directed, weighted edges. The weights, termed *h* values, are stored in the so-called *h* matrix and in turn determine the transition probability from one clip to another. The deliberation process is thus modelled as a random walk through the clip network. The ECM has a flexible structure that may consist of several layers and that can change over time by, for instance, the creation of new clips and their addition to the existing network (see e.g. [41]). However, for the purpose of this work, it is sufficient to consider the basic two-layered structure (see Fig. 2), where one layer of *percept-clips* (or just “percepts”, for brevity) encodes the perceptual information that the agent gets from its surroundings, and another layer of *action-clips* (or just “actions”) encodes the information about the possible actions the agent can take.

The interaction round of an individual PS agent goes as follows: first, it perceives certain input information that activates the corresponding percept-clip in the ECM, which triggers a random walk through the network that ends when an action-clip, and subsequently its corresponding actuator, are activated, leading the agent to actually perform the action. Therefore, the final action that the agent will execute depends on the transition probabilities from clip to clip in the ECM, which are determined by the *h* values as,

1 $$ p_{{ij}}=\frac{h_{{ij}}}{\sum_{k} h_{{ik}}},   $$

where the transition probability from clip *i* to clip *j*, *p*_*ij*_, is given by the weight *h*_*ij*_ of the edge that connects them, normalised over all the possible outgoing transitions to clips *k* connected to *i*. In this work, we consider the two-layered PS, where each percept clip is only connected to all the action clips, so *p*_*ij*_ is simply the transition probability from percept *i* to action *j*.

A reinforcement learning mechanism can be implemented by updating the *h* values at the end of an interaction round. If the agent’s choice is good, it receives a reward *R* that increases the *h* value of the traversed percept-action edge, so that the agent has higher probability of performing that action the next time the same percept clip is activated. At the beginning of the learning process, we consider that the agent chooses one of the possible actions at random. Therefore, all edges are initialised with the same *h* value, $h_{{ij}}^{(0)}=1$, which leads to a uniform probability distribution over the actions. In addition to an increase of the edge weights throughout the learning process, noise can be added by introducing a *forgetting* parameter *γ* (0≤*γ*<1) that quantifies how much the *h* values are damped towards their initial value. This can be interpreted as the agent forgetting part of its past experience. The specific update rule at the end of the interaction round for an edge connecting percept *i* to action *j* has the form,

2 $$ h_{{ij}}^{(t+1)} \longleftarrow h_{{ij}}^{(t)} - \gamma (h_{{ij}}^{(t)}-h_{{ij}}^{(0)}) + R,   $$

where $h_{{ij}}^{(t)}$ denotes the current *h* value, $h_{{ij}}^{(0)}$ the initialised *h* value at the beginning of the learning process and *R*≥0 the reward. If the transition from percept *i* to action *j* is rewarded, then *R* has a value *R*>0, whereas if it is not rewarded, *R*=0 and the edge weight is only damped. Note that this update rule increases the *h* value at the end of each interaction round, depending on whether a reward is given for that round. If one considers a scenario where the agent interacts for several rounds and only gets a reward at the end of the last one, then only the percept-action edge that is traversed in that round is enhanced. In order to reinforce all the percept-action pairs that led to a reward, a mechanism called *glow* is introduced as part of the model. The idea is to keep track of which edges are traversed during the interaction rounds before the reward is given. To do so, once an edge is traversed, a certain level of excitation or “glow” is associated to it with the effect that, when the *next* reward is given, all the “glowing” edges are enhanced in proportion to their glow level. The glow for each edge *i*→*j* is stored in the element *g*_*ij*_ of the so-called glow matrix *g*, and the update rule of Eq. (2) takes the form,

3 $$ h_{{ij}}^{(t+1)} \longleftarrow h_{{ij}}^{(t)} - \gamma (h_{{ij}}^{(t)}-h_{{ij}}^{(0)}) + g_{{ij}} R.   $$

Therefore, if that edge is glowing (*g*_*ij*_=1) at the end of the interaction round where the reward is given, it will be enhanced. For the purpose of this work, it is sufficient for the reader to consider that edges get a glow value *g*_*ij*_=1 if they are traversed and *g*_*ij*_=0 if they are not. For more details on how to assign and update glow values in different scenarios, we refer the reader to [38].

So far, we have described the main processes that a single PS agent carries out when interacting with its surroundings and learning via reinforcement. In this work, we model a scenario with an ensemble of PS agents, each of which has its own deliberation process and makes decisions independently from the rest of the ensemble. The precise details of how the agents interact with each other and how the collective performance is evaluated are given in the “Details of the model I: the bee and the learning process” section. There are a few remarks still to be made regarding the learning process of such an ensemble and its interpretation from a biological point of view. In this work, we do not assume that the individual biological entities have the capacity to learn, but we consider the learning process from an evolutionary perspective. Hence, the improvement of the collective performance of the ensemble of agents throughout the learning process can be interpreted as the adaptation of a given species to certain pressures throughout its entire evolutionary history. In the context of reinforcement learning, the selection pressure is encoded in the reward function, in such a way that we can test hypotheses about which environmental factors may have influenced the resultant behaviour we currently observe in the real organisms. In this view, the forgetting parameter would capture one aspect of genetic drift.

### Details of the model I: the bee and the learning process

We consider a population of *N* bees, where each bee is modelled as a PS agent that perceives, decides, acts and learns according to the model explained in the previous section. Unless specified otherwise, we always use *N*=100. The population is confronted with the pressure of a predator that attacks the colony and kills agents until it is scared away, i.e. until it is stung a certain number of times. In this section, we describe how we model the colony and the learning process. We give further details on the model of the predator in the “Details of the model II: the predator” section.

Honeybees release an alarm pheromone when their stinger is exposed, which allows them to alert and recruit nearby bees into a collective defensive response. Since we are interested in explaining the experimentally observed response of bees to the sting alarm pheromone, we consider that the agents decide whether to sting or not based on the pheromone concentration they perceive. The alarm pheromone concentration is discretised in our model and it increases by one unit every time an agent decides to sting. This is the only mechanism for SAP release that we consider. The honeybee SAP disperses very fast when the stinger is extruded [1], so we considered this increase to be immediate. In addition, this pheromone has been shown to accumulate within the time-frame relevant for an attack [44]. Hence, the minimum concentration is 0 units, when no agent stings, and the maximum is *N* units, if all agents sting. Note that each agent can only sting once, like a real bee (when a bee stings an animal with elastic skin such as a mammal or bird, its stinger remains hooked into the predator thanks to its barbs, and tears loose from the bee’s abdomen).

In order to define the structure of the ECM and the percepts of the PS model, we group the range of *N* pheromone units into bins of logarithmic size, where each bin corresponds to one percept (see Fig. 3). There are two reasons for this choice, exposed thereafter. Nonetheless, we also explored other types of binning, as presented in Additional file 2. First, the logarithmic sensing implies that the agents are able to resolve with high precision low values of pheromone concentration but that precision decreases as the concentration increases. On the one hand, with a range of pheromone units extending to *N*∼10^2^, it is plausible to assume that the agent can distinguish between 0 and 1 pheromone units but a change by 1 pheromone unit becomes harder to sense when the concentration is of the order of e.g. 30 units. Furthermore, several species of animals present a logarithmic relationship between the stimuli and their perception, as it is the case for instance with human perception, which is quantified by the Weber-Fechner law. The second reason for this choice is related to the structure of the PS agents’ interaction. We consider the collective interaction to be sequential, i.e. the agents perceive, decide and act one after the other until all of the *N* agents have made their decision and acted. This a priori artificial condition becomes more realistic with the logarithmic binning since several agents perceive the same percept and hence act based on the same input information, which is effectively as if they would react in parallel. An illustrative example of the sequential interaction, named trial, is given in Fig. 3. Each trial is always a defensive event that is triggered by an unspecified disturbance outside the nest. Each of the agents decides only once whether to sting or not; hence, the trial comprises *N* time steps, where a “time step” is defined to be the decision time of one agent.

Since we want to model a situation as close as possible to the experimental one, we assume that our agents also have a visual perception of the predator during the whole process (the bees see a rotating dummy, as explained in the “Experimental material and methods” section). As in the experimental setup, we consider that this visual perception remains unchanged while the agent perceives the increase in the pheromone concentration (percepts 0 to 8, see Fig. 3). However, in our model, we add an additional percept (labelled as *v*_ESC_ in Fig. 3) that is activated when the visual perception of the predator changes and the agents *see* that the predator is already escaping and is no longer a threat to the colony. In this case, we assume that the visual input overrides the olfactory one so that the agents decide based on the visual information only. This is consistent with reports that show that while the alarm pheromone recruits bees to the location of the disturbance, a moving visual stimulus is then necessary to release the stinging behaviour [45]. In order to study the interplay between the visual and the olfactory information and their influence on the self-limitation of the stinging response, we consider the percept *v*_ESC_ to not be activated immediately after the predator stops attacking, but after some time delay *Δt*_*v*_. This time delay is more realistic than an immediate cut-off: it corresponds to the time needed for the bees to perceive that the predator is retreating. In addition, the predator may need time to move outside of the perimeter defended by the resident bees.

In order to avoid unnecessary losses, we could expect the agents to learn to stop stinging already during the interval *Δt*_*v*_, based on the pheromone concentration (i.e. on the number of bees that already stung, which may be sufficient to deter any predator). We can thus analyse the two extreme cases with *Δt*_*v*_=0 and *Δt*_*v*_=*∞*, which correspond respectively to when bees are able to immediately distinguish that the predator is leaving (*Δt*_*v*_=0) or to when they completely ignore this visual cue (*Δt*_*v*_=*∞*). In the former case, we expect no self-limiting mechanism to develop, since the end of the attack is always immediately signalled by the visual stimulus. In the latter scenario however, unusually high alarm pheromone concentrations may start playing the role of this “stop signal”. Note that in the experimental setup the dummy is always rotating; hence, the bees’ reactions are modulated by the pheromone level exclusively.

With respect to the learning process, the performance of the population is evaluated at the end of one sequential interaction (or *trial*) and the individuals are rewarded or not depending on the final state of the colony. Thus, the agents’ ECMs do not change during the trial. At the end of it, a reward that is proportional to the number of surviving agents is given and the ECMs of the agents are updated accordingly. Importantly, the same *h* matrix is used for every agent of the population. The reason for this choice is that genetic mixing during reproduction means that, effectively, the average individual responses to the alarm pheromone are transmitted to the next generation [46]. One could easily imagine that a scenario in which certain agents always sting while others never do may lead to a viable collective strategy, but such a population structure could not be stably maintained across generations. As explained in the “Theoretical model: Projective Simulation” section, the update rule of Eq. (3), now given in matrix form, reads,

4 $$ h^{(t+1)}=h^{(t)}-\gamma \left(h^{(t)}-h^{(0)}\right) + g R.  $$

where *h*^(*t*)^ is the *h* matrix at the current trial and *h*^(0)^ denotes the initial *h* matrix (a 2×10 matrix with ones in all its entries). Note that agents start with a probability of stinging *p*_*s*_=0.5 for all the percepts. The already learned responses are damped by a factor *γ* at the end of each trial. The influence of this parameter on the learning process is further studied in Additional file 2. In this work, we adapt the notion of a glow matrix *g* presented in the “Theoretical model: Projective Simulation” section to take into account the choices of all agents and distribute the reward depending not on the individual performance but on the collective one. We remark that the learning process is, in our case, interpreted as the evolutionary history of honeybees. Therefore, even though there exist fluctuations at the individual level, we are interested in the average effect on the population. From this perspective, we consider a glow matrix *g* that stores how many agents chose an action given a certain percept. In the example of Fig. 3, the second column of *g* — the one corresponding to percept 1 — in that trial is (9,3), which indicates that 9 agents decided not to sting and 3 decided to sting. If the population is rewarded at the end of the trial, the individual responses that lead to a good collective performance are enhanced. For instance, if the optimal defence is that all the bees sting from the beginning, the individual probability of stinging for low pheromone concentrations will converge to a high value.

As to the reward, it is determined by the percentage of bees that remain alive at the end of the trial,

5 $$ R=\frac{a}{N},   $$

where *a* denotes the number of live bees. This number is evaluated at the end of each trial, by subtracting the number of dead bees from the total number of agents *N*. Bees die after stinging (*s*) or because they are killed by the predator (*q*),

6 $$ a=N-s-q.  $$

Note that the number of agents does not change during the trial (all *N* agents get to decide and act, bees killed by the predator are only counted at the end of the trial). This choice of reward system reflects the fact that honeybee colonies with a larger workforce are more successful ecologically: they have a better chance of surviving winter [47], and most importantly, they are more likely to be able to invest in reproduction in spring [48]. The reward function presented here is linear. We also explored a different scaling of this linear function and non-linear functions, and the results are included in Additional file 3: Figure S3.

In the simulations reported here, the entire learning process consists of 80,000 trials, which is sufficient for the population to converge to a stable behaviour.

We remark that the learning processes that the populations of PS agents undergo are interpreted as processes of adaptation to given evolutionary pressures. In this work, we focus on the defence behaviour against predators, so, by changing the parameters of our predator model, we are effectively testing how different pressures affect the final behaviour. This allows us to analyse possible causal explanations for the responses observed in present-day real bees.

### Details of the model II: the predator

The predator has an active role in our model, since it attacks the hive and kills bees at a given killing rate of *k* bees per time step (for an exploration of the role of this parameter, see Additional file 3: Figure S4). Therefore, the colony needs to build up the defence as fast as possible to reduce the number of bees killed by the predator. Of particular importance is the time needed for the bees to detect the presence of the predator, which we parametrise as the time step at which the predator starts its attack *t*_*att*_ (see Fig. 3). A low value for this parameter simulates cases in which the predator is only detected close to the colony and hence starts killing bees quickly. At the opposite, a high value for *t*_*att*_ represents an early detection by the bees, when they have more time to fly out and build up the defensive response before the predator reaches the nest itself.

The predator stops killing bees when the number of total stings reaches a threshold *s*_*th*_. By changing this parameter, we model the type of predator that the colony may encounter. As an example, one may consider that small predators such as mice can be killed or scared away with fewer stings than bigger or thick-skinned animals like bears and honey badgers. Thus, different *s*_*th*_ can be interpreted as differences in the predator’s resistance to bee stings.

In the wild, bees regularly encounter a wide variety of predators, and they need to be able to cope with all of them. We model this situation by introducing a range of *s*_*th*_. Instead of being faced with only one type of predator (same *s*_*th*_ in all trials), the colony is attacked by a predator with a different *s*_*th*_ for different trials, which is chosen from a uniform distribution over a certain range. Therefore, the parameter *s*_*th*_ gives us the flexibility to model different environmental conditions. For instance, we can model colonies of bees that are usually attacked by small/less resistant predators and observe the defensive strategy that they adopt. We can then study how they respond when suddenly faced with bigger/more resilient predators, thus mimicking their introduction into a novel environment.

Since the agents can only develop one strategy (i.e. a set of probabilities of stinging for the various percepts) per learning process, they have to optimise it to accommodate the whole range of *s*_*th*_. Note that the activation of the visual percept *v*_ESC_ — which happens when the visual information changes and the predator is seen leaving — allows them to stop the defence behaviour at different points of the trial depending on the specific *s*_*th*_ of each predator. In particular, percept *v*_ESC_ is activated after *Δt*_*v*_ time steps from the point where the number of stings reaches the corresponding *s*_*th*_.

## Supplementary Information

**Additional file 1** Learned probabilities of stinging and total number of stings for the limiting cases with *Δt*_*v*_=0,*∞*.

**Additional file 2** Study of the influence of the forgetting parameter (hyperparameter optimisation) and the sensing resolution in the learning process.

**Additional file 3** Study of the influence of the reward function used and of the killing rate.

## Data Availability

The theoretical results were obtained using the code CollectiveStinging, available at https://github.com/qic-ibk/CollectiveStinging. The experimental data are available upon request to MN (morgane.nouvian@uni-konstanz.de). Declarations

## Abbreviations

ECM: Episodic and compositional memory
IAA: Isoamyl acetate
PID: Photoionization detector
PS: Projective Simulation
SAP: Sting alarm pheromone
