# Supplementary material for: Honeybee communication during collective defence is shaped by predation
Source: BMC Biol. 2021 May 25;19:106. doi: 10.1186/s12915-021-01028-x (PMC8147350; doi:10.1186/s12915-021-01028-x)
Supplement: Supplementary file 1 — Additional file 1 Learned probabilities of stinging and total number of stings for the limiting cases with Δtv=0,∞. [file 12915_2021_1028_MOESM1_ESM.pdf]

Honeybee communication during collective defence is shaped by predation

Additional file 1: Figure S1

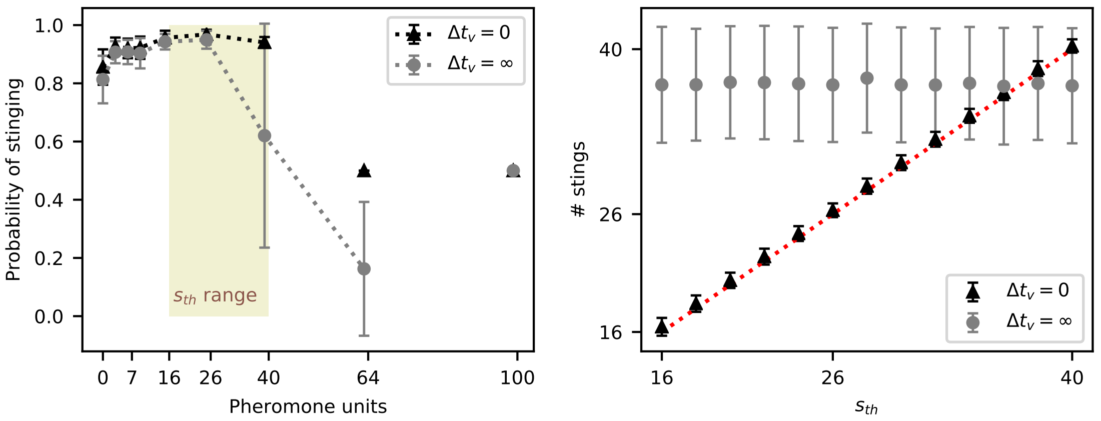

Figure S1: Learned probabilities of stinging and total number of stings for the limiting cases with $\Delta t_v = 0, \infty$. Colonies that are able to visually detect that the predator is escaping immediately after s_{th} is reached ($\Delta t_v = 0$) sting with high probability for all the pheromone concentrations they perceive (values of p_s that remain as initialised are not connected by dotted line). The observed decay is a return to the baseline values of $p_s = 0.5$. The probability of stinging for percept v_{ESC} is $p_s = 0.005 \pm 0.003$. The bees sting until v_{ESC} is activated (red dotted line on the right panel). Colonies with no visual signal ($\Delta t_v = \infty$) rely on the information about the alarm pheromone concentration to stop stinging. The decay part of the curve in this case indicates a self-limiting mechanism (last percept is never activated, so its value remains as initialised $p_s = 0.5$). These colonies sting the same number of times for all types of predators (right panel). Average is obtained by taking data from 50 independently trained ensembles of 100 agents. For the right panel, the performance on the last 500 trials of the learning process is considered. Predator's parameters: $s_{th} \in (16, 40), t_{att} = 0, k = 1, r_f = 0$ (no false alarms). Forgetting: $\gamma = 0.003$.
