# Supplementary material for: Honeybee communication during collective defence is shaped by predation
Source: BMC Biol. 2021 May 25;19:106. doi: 10.1186/s12915-021-01028-x (PMC8147350; doi:10.1186/s12915-021-01028-x)
Supplement: Supplementary file 2 — Additional file 2 Study of the influence of the forgetting parameter (hyperparameter optimisation) and the sensing resolution in the learning process. [file 12915_2021_1028_MOESM2_ESM.pdf]

Honeybee communication during collective defence is shaped by predation

Additional file 2: Calibration of the learning process

Before PS could be successfully applied to our model, we determined which learning parameters — also called hyperparameters— should be selected in order to optimize the model's global performance [1]. In particular, we investigated the influence of the forgetting parameter (γ).

To this end, we first set up a task admitting a clear optimal collective strategy that could be easily predicted beforehand. We considered just one predator with $s_{th} = 26$, $t_{att} = 0$ and $\Delta t_v = 10$, for which the optimal strategy is known: the first 26 bees should sting with $p_s = 1$ and the remaining ones should not sting ($p_s = 0$). Given a killing rate $k = 1$, the number of live bees at the end of the trial has a theoretical maximum of $a^* = 100 - 2 \cdot 26 = 48$. Note that we have chosen $s_{th} = 26$ so as to allow the bees to distinguish when they have stung exactly 26 times, since this is the start of a new binning interval that corresponds to a different percept (percept 6). Fig. S2a shows the learned probability of stinging as a function of the pheromone units for cases with $\gamma = 0, 0.001, 0.003, 0.01$. The behaviour that is closest to the optimal one is obtained for γ with order of magnitude 10^{-3}. Indeed, if the forgetting is too low ($\gamma = 0$) or too high ($\gamma = 0.01$), the agents' behaviour converged quickly (as can be seen on Fig. S2b) but got stuck in local minima, and the optimal performance a^* was not reached. Looking at the strategy adopted by these populations, the probability of stinging at low pheromone concentrations appeared lower, which lead to a slower collective defensive response, allowing the predator to kill more bees before it is scared away. Note that the last two percepts, corresponding to pheromone units up to 63 and 99, respectively, retained the initial values $p_s = 0.5$. This is due to the fact that, once agents learned to stop stinging at percept 6 (26-39 pheromone units), they did not encounter those two percepts any more so their values decayed back to 0.5. Thus these two probabilities did not affect the final collective performance at all. As expected, the agents also learned to stop stinging when the percept v_{ESC} was activated. The obtained values are $p_s = 0.25 \pm 0.03$ for $\gamma = 0$, $p_s = 0.042 \pm 0.007$ for $\gamma = 0.001$, $p_s = 0.006 \pm 0.003$ for $\gamma = 0.003$ and $p_s = 0.015 \pm 0.007$ for $\gamma = 0.01$. Note that populations trained with γ in the range of 10^{-3} again achieved the lowest value, which further supports our finding that this order of magnitude for the forgetting parameter is suitable.

The forgetting parameter is also crucial in tasks with changing objectives, for instance when different predators attack the colony (i.e. when s_{th} can vary throughout the trial). To refine our calibration of γ, we thus considered a second task in which s_{th} was chosen from a uniform distribution over the range $s_{th} \in (16, 40)$. In this case the agents need to develop a single strategy that is suitable for all values of s_{th} within the same learning process, that is, there is no independent learning process for different values of s_{th}. Based on our previous results, we varied γ systematically in the range $(0.001, 0.1)$ in order to select the forgetting parameter that lead to the best performance. The results are presented in the inset of Fig. S2c, where we see that the value $\gamma = 0.003$ achieved the best performance. Therefore, we chose this value to further extend our analysis and study the influence

of the predator's parameters in the main text. As examples, Fig. S2c shows the learned probabilities of stinging for processes with $\gamma = 0.001, 0.003, 0.007$. We observe that p_s has a high value for pheromone concentrations below 26 pheromone units. At higher concentrations, the probability of stinging decreases gradually, reflecting the fact that only few predators necessitate that many stings before escaping. By contrast, when only one value of s_{th} was considered in the learning process, the decrease of p_s was more abrupt (see Fig. S2a). Finally, the agents learned to stop stinging when the percept v_{ESC} was activated in all three learning processes, with $p_s = 0.039 \pm 0.007$ for $\gamma = 0.001$, $p_s = 0.005 \pm 0.002$ for $\gamma = 0.003$ and $p_s = 0.012 \pm 0.005$ for $\gamma = 0.007$. The lowest value of p_s was again achieved for $\gamma = 0.003$, thus further validating our choice.

Another important choice we had to make was to set the number of percepts in the bee's ECM. Here, we analysed the influence of this parameter and of the logarithmic binning, in order to check if different choices in parametrizing the bees' perception could influence the strategy adopted by the colony. To do so, we considered a learning process with a range of predators $s_{th} \in (16, 40)$ and $\gamma = 0.003$ and we varied the resolution with which the agents could perceive the number of pheromone units. Fig. S2d shows the learned probabilities of stinging for processes with 9 and 21 percepts, both with logarithmic binning, and with 100 percepts (full resolution, linear/no binning since the agents are able to resolve every single pheromone unit). Note that, for the logarithmic binning, we considered that the minimum size of each bin is three pheromone units. We observe that the behaviour adopted by the agents did not qualitatively change depending on our choices in modelling their perceptual mechanisms. In all three processes, agents learned to sting with high probability when the concentration of pheromone was low and to stop stinging around 40 units, which is the s_{th} of the largest predator. As expected the probability of stinging for percept v_{ESC} tended to zero in all cases: $p_s = 0.005 \pm 0.002$, $p_s = 0.006 \pm 0.003$ and $p_s = 0.006 \pm 0.002$, for processes with 9, 21 and 100 percepts respectively. The only difference between these 3 ways of modelling the sensory perception of the agents was the resolution achieved: more percepts (i.e. smaller bins) allowed more gradual transitions in p_s values. Therefore, in the rest of the study we considered only 9 percepts, as this made the learning process more stable and less computationally demanding.

References

[1] Alexey A Melnikov, Adi Makmal, and Hans J Briegel. Benchmarking projective simulation in navigation problems. *IEEE Access*, 6:64639–64648, 2018.

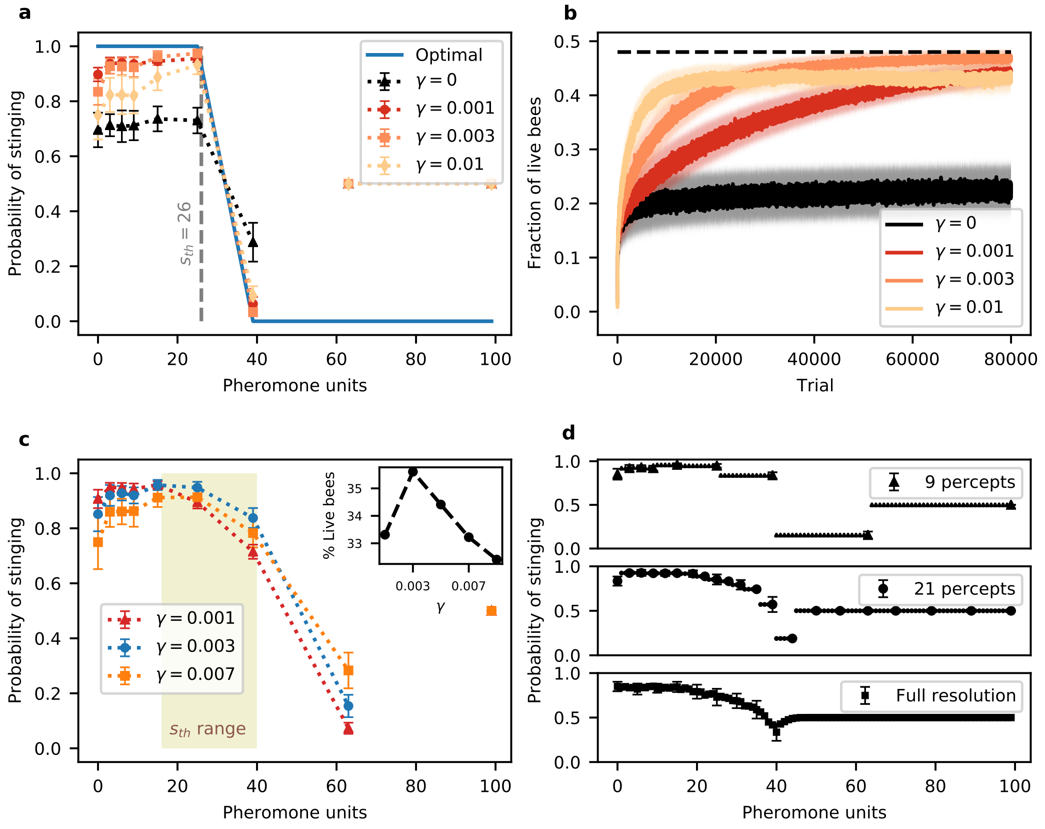

Figure S2: (a) Calibration of the learning process with respect to the forgetting parameter γ. A scenario where the optimal response is known in advance is considered. Specifically, one predator with $s_{th} = 26$ and $k = 1$ attacks the colony at $t_{att} = 0$ ($\Delta t_v = 10$), so the best collective strategy is to sting with probability $p_s = 1$ until the concentration of alarm pheromone reaches 26 units and then stop ($p_s = 0$). The values of γ that achieve the closest responses to the optimal ones are of the order 10^{-3}. Markers indicate the upper end of each perceptual bin. (b) Evolution of the fraction of live bees throughout the same learning processes. The dashed line indicates the optimal value of $100 - 2 \times 26 = 48$ live bees. Shaded areas indicate one standard deviation. (c) Influence of γ in a learning process with a range of predators $s_{th} \in (16, 40)$ ($k = 1, t_{att} = 0, \Delta t_v = 10$). Learned probabilities for processes with $\gamma = 0.001, 0.003, 0.007$. The inset shows that the value 0.003 achieves the best performance of values in the range $(0.001, 0.01)$. (d) With γ fixed at 0.003, an increase in the sensing resolution does not qualitatively change the learned responses. "Full resolution" denotes that agents are able to resolve an increase in one pheromone unit, whereas in the other two learning processes, agents have logarithmic sensing with 9 and 21 percepts, respectively. Large markers are at the end of each percept's range of pheromone units. In all panels, averages are obtained by taking data of 50 independently trained ensembles of 100 agents.
