# Supplementary material for: Honeybee communication during collective defence is shaped by predation
Source: BMC Biol. 2021 May 25;19:106. doi: 10.1186/s12915-021-01028-x (PMC8147350; doi:10.1186/s12915-021-01028-x)
Supplement: Supplementary file 3 — Additional file 3 Study of the influence of the reward function used and of the killing rate. [file 12915_2021_1028_MOESM3_ESM.pdf]

Honeybee communication during collective defence is shaped by predation

Additional file 3: Figure S3, Figure S4

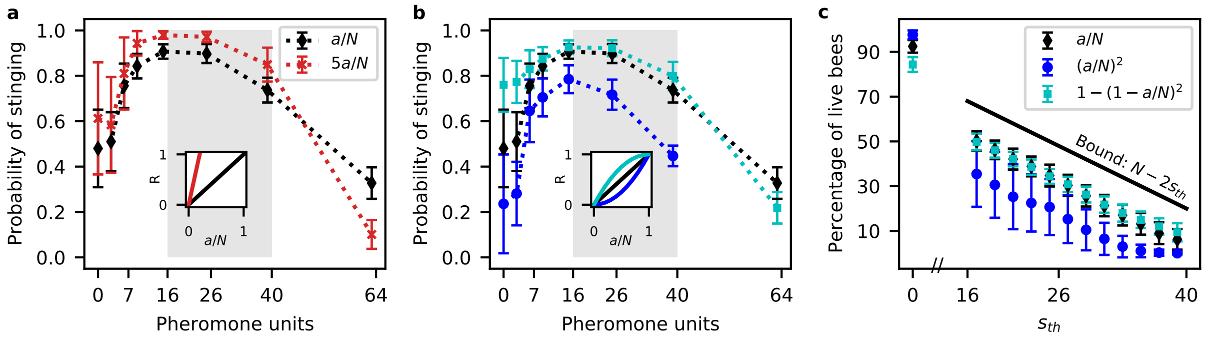

Figure S3: Influence of the reward function on the responsiveness to the alarm pheromone (data from modelling). (a) Scaling effect on a linear reward function. Colonies that have a higher individual life cost (modelled by re-scaling the reward function to $R = 5a/N$ instead of $R = a/N$) develop more extreme reactions to the alarm pheromone. They sting with higher probability at lower concentrations but with lower probability at the highest concentrations (stronger self-limitation). (b,c) Comparison between linear and quadratic reward functions. A linear reward function considers that each bee's life has the same value for the colony, which is not the case when considering quadratic functions. With the reward function $R = 1 - (1 - a/N)^2$ (light blue), the value of a bee's life increases as the colony becomes smaller. With $R = (a/N)^2$, (dark blue) this cost is larger when the colony is still populous, which is maybe less realistic. Our results indicate that the populations trained with $R = 1 - (1 - a/N)^2$ perform better against resistant predators, but are more prone to wasting bees during false alarms. Populations trained with $R = (a/N)^2$ have an opposite pattern (they avoid false alarms more successfully, but are not as efficient against resistant predators). The linear reward function $R = a/N$ leads to a more balanced performance in both scenarios. When the reward function is $R = (a/N)^2$, there is also a higher variability in the learned responses to low concentrations between populations, which in turn leads to variability in their performance especially against weak predators (see std in panel c). In panels a and b, we plot the average response ± one standard deviation of 50 independently trained populations of 100 agents. The insets show the respective reward functions. Points for which p_s remains as initialised are not shown. Shaded areas correspond to the s_{th} range. Probability of stinging for visual percept v_{ESC}: $p_s = 0.024 \pm 0.010$ (for $R = a/N$), $p_s = 0.024 \pm 0.009$ (for $R = 5a/N$), $p_s = 0.085 \pm 0.016$ (for $R = 1 - (1 - a/N)^2$), $p_s = 0.006 \pm 0.003$ (for $R = (a/N)^2$). (c) Average ± one standard deviation in the last 500 trials of 50 independently trained populations. For all panels, the predator's parameters are: $s_{th} \in (16, 40), k = 1, t_{att} = 0, \Delta t_v = 10, r_f = 0.6$. Forgetting: $\gamma = 0.003$.

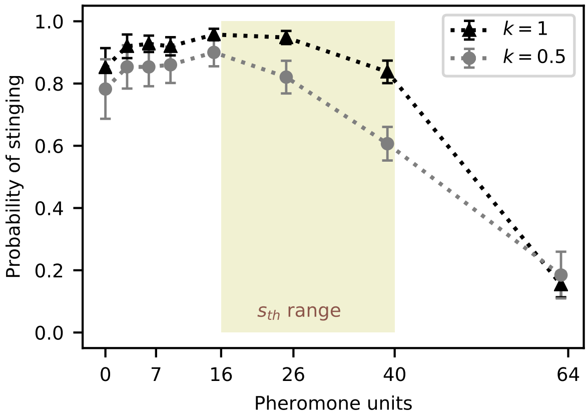

Figure S4: Stinging response as a function of alarm pheromone concentration for populations facing predators with different killing rates (data from modelling). For all populations, the predator parameters are $s_{th} \in (16, 40)$, $t_{att} = 0$, $r_f = 0$ and $\Delta t_v = 10$. The killing rate is varied such that $k = 1$ for some populations (black triangles) and $k = 0.5$ for others (grey circles). Populations facing less damaging predators develop a weaker response to the alarm pheromone, especially at high concentrations. Visual percept (for $k = 0.5$): $p_s(v_{\mathrm{ESC}}) = 0.008 \pm 0.005$. Averages $\pm$ one standard deviation, obtained by taking data over 50 independently trained populations of 100 agents. Forgetting: $\gamma = 0.003$.
